# Induction of α-amylase and endosperm-imposed seed dormancy: two pioneering papers in gibberellin research

**DOI:** 10.1007/s00425-025-04699-w

**Published:** 2025-04-25

**Authors:** Peter Hedden

**Affiliations:** 1 Laboratory of Growth Regulators, Institute of Experimental Botany and Palacký University Olomouc, 78371 Olomouc, Czech Republic; 2 Sustainable Soils and Crops, Rothamsted Research, Harpenden, AL5 2 JQ UK

**Keywords:** α-amylase, Aleurone, Bioassays, Endosperm, Germination, Gibberellin action

## Abstract

**Main conclusion:**

Two papers with quite different objectives established protocols that proved pivotal for future work on the role of gibberellins in seed germination.

**Abstract:**

In their paper published in 1967, Russell Jones and Joseph Varner (Planta 72: 155–161) developed a bioassay based on induction of α-amylase activity in barley embryo-less half-seeds that was specific for bioactive gibberellins. The induction of α-amylase in the aleurone of barley and other cereals was to become the experimental system of choice to study gibberellin signalling. However, despite much progress in identifying the molecular events linking gibberellin action and α-amylase gene expression, in many cases their role in the process is still unclear. In 1987, Steven Groot and Cees Karssen (Planta 171:525–531) showed that germination of tomato seeds was limited by the ability of the radicle to penetrate the surrounding layers, with the endosperm forming the major barrier. They used a modified needle attached to a tensiometer to measure the force required to break through the endosperm. While in wild-type seeds, a factor from the embryo, assumed to be gibberellin, promoted breakdown of the endosperm, gibberellin-deficient seeds required an external supply of the hormone to weaken the endosperm or for it to be mechanically disrupted for germination to occur. The paradigm of seed germination being physically restricted by surrounding layers and the role of gibberellin in weakening these tissues has been confirmed in many eudicot species. Gibberellin signalling induces the production of cell-wall loosening enzymes in the micropylar endosperm adjacent to the radicle, but it is unclear whether or not this is a direct response. In both eudicot and monocot systems, there is still much to learn about the role of gibberellin signalling in germination.

## Introduction

Two pioneering papers published in Planta 20 years apart and with different aims addressed the role of gibberellins (GAs) in seed germination (Groot and Karssen 1987; Jones and Varner 1966). Gibberellins are known to be major players in promoting seed germination and the molecular mechanisms by which they regulate this process were to become major topics in plant research (Gong et al. 2022; Peng and Harberd 2002; Nonogaki 2014). Both papers introduced novel experimental protocols which formed springboards for future advances in their respective areas.

The paper by Russell Jones and Joseph Varner entitled “The bioassay of gibberellins” published in 1967 described the exploitation of α-amylase production by germinating barley (*Hordeum vulgare*) seeds for the development of a highly specific bioassay for GAs. The paper was identified as a Citation Classic in 1986, by which time it had been cited 215 times (Jones 1985). The molecular mechanism by which GAs induce the synthesis of α-amylase and other hydrolytic enzymes in cereal aleurone became an active area of research and one of the first systems to be used to investigate GA signalling (Bethke et al. 1997; Lovegrove and Hooley 2000; Hedden and Sponsel 2015).

The second paper by Steven Groot and Cees Karssen entitled “Gibberellins regulate seed germination in tomato by endosperm weakening: a study with gibberellin-deficient mutants” was published in 1987. It describes early events in GA-induced seed germination in this species and, as evidenced by its current 291 citations (World of Science), was an influential forerunner of numerous papers on the physical, physiological and biochemical events required for this process (Nonogaki 2019).

### A specific and reliable bioassay for gibberellins

In the absence of physicochemical methods to analyse GAs, early assays for these hormones were based on their ability to stimulate tissue growth, particularly in GA-deficient dwarf mutants (Brian et al. 1962). The discovery that GA induced α-amylase production in barley aleurone (Paleg 1960; Varner and Ram Chandra 1964; Yomo 1960) provided on opportunity for a novel bioassay that was highly specific for GAs. The original assays measured the release of reducing sugars from starch due to the action of α-amylase, with the activity proportional to the logarithm of GA_3_ concentration (Nicholls and Paleg 1963). However, as pointed out by Jones and Varner, the assay required the measurement of reducing activity and was susceptible to interference, for example by impurities in the solvents used to extract GAs. In Jones and Varner’s assay, amylase activity was measured more directly from the breakdown of starch determined using iodine reagent (I_2_/KI). Sterilised barley half-seeds, without the embryo, were imbibed for three days and then incubated in buffer with the test solution for a further 24 h. After centrifugation, α-amylase activity was measured in the supernatant after addition of potato starch and the iodine reagent. The assay gave a linear response between 10^–9^ and 10^–7^ M GA_3_ on a logarithmic scale, proved well-suited to analysing fractions from plant extracts and was not activated by solvent impurities. In a comparison of GA_3_ with GAs 1, 4, 5 and 7, GA_3_ was the most active, although, with the exception of GA_5_, the other GAs were also highly active. Although not tested in this study, subsequent publications, for example, Crozier et al. (1970) have shown that the barley α-amylase assay is specific for bioactive GAs since the aleurone is not capable of converting biosynthetic precursors into active forms. Amylase production is, however, inhibited by abscisic acid (ABA).

The α-amylase bioassay was used and adapted in numerous studies as a means to detect and quantify “GA-like substances”, but it and other bioassays were superseded by physicochemical methods, initially combined gas chromatography-mass spectrometry (GC–MS) (Binks et al. 1969), and immunoassays (Yamaguchi and Weiler 1991), which offered better molecular specificity. It should be noted, however, that the specificity of immunoassays depends on that of the antibodies and none were completely specific for single GA forms. GC–MS proved valuable for identification and, in combination with isotopically labelled internal standards, for quantification of GAs and their biosynthetic precursors and catabolites (Croker et al. 1994). The method has now been largely replaced by liquid chromatography–mass chromatography (LC–MS), which requires no derivatization and less stringent pre-purification, although derivatization of GAs has been used to improve mass spectrometric sensitivity by enabling positive ion detection (Deng et al. 2017; Kojima et al. 2009). Ultra-performance LC–MS (UPLC-MS), which offers analyte separation with high resolution, has become a standard method for the quantitative analysis of GAs and other plant hormones in many laboratories. The reliability of this method is dependent on adequate sample purification to reduce the chemical background, the use of appropriate internal standards and due care with data evaluation. Otherwise, the method can produce misleading information and in fact be less reliable than bioassays at estimating GA levels. UPLC coupled with tandem mass spectrometric methods such as multiple reaction monitoring offers high selectivity and is a very powerful technique (Urbanova et al. 2013), but without the appropriate care there is a danger that GA analysis may have regressed from the era of bioassays.

The sensitivity of UPLC-MS is increasing with improved technology, but is still not sufficient to analyse GAs in plant organs with high spatial resolution. GAs are not distributed uniformly and a better understanding of GA function requires knowledge of their distribution, ideally at the cell level (Binenbaum et al. 2018). This is being addressed by the use of in situ methods such as the Gibberellin Perception Sensors (GPS), based on the GA receptor, that allow bioactive GAs to be visualized using FRET (Griffiths et al. 2024; Rizza et al. 2017). GPS measures relative GA levels, rather than absolute concentrations, at cellular resolution so is ideal for determining GA distribution and gradients. However, this method is currently limited to bioactive GAs, which bind to the receptor, while information on the distribution of biosynthetic precursors and catabolites is needed to understand how GA concentration is regulated.

### Gibberellin signalling and the expression of α-amylase genes in the cereal aleurone

The mechanism by which GA promotes and ABA suppresses α-amylase gene expression and its involvement in germination of cereal grains has been an active research area. The aleurone provided an ideal system in which to study GA signal transduction since it is dependent on an external source of GA, provides a uniform cell population and is amenable to the preparation of protoplasts that retain their response to GA (Hedden and Sponsel 2015). However, although knowledge in this area has advanced considerably, the full mechanism is still unclear and work on this topic has waned somewhat in recent years. There were high hopes that the cereal aleurone would yield the elusive GA receptor, and experiments with this system suggested it was located on the plasma membrane (Bethke et al. 1997). However, with the discovery that GAs were perceived by a soluble nuclear receptor GIBBERELLIN INSENSITIVE DWARF 1 (GID1) (Ueguchi-Tanaka et al. 2005), there was reduced interest in the cereal aleurone as an experimental tool.

**Fig. 1:**
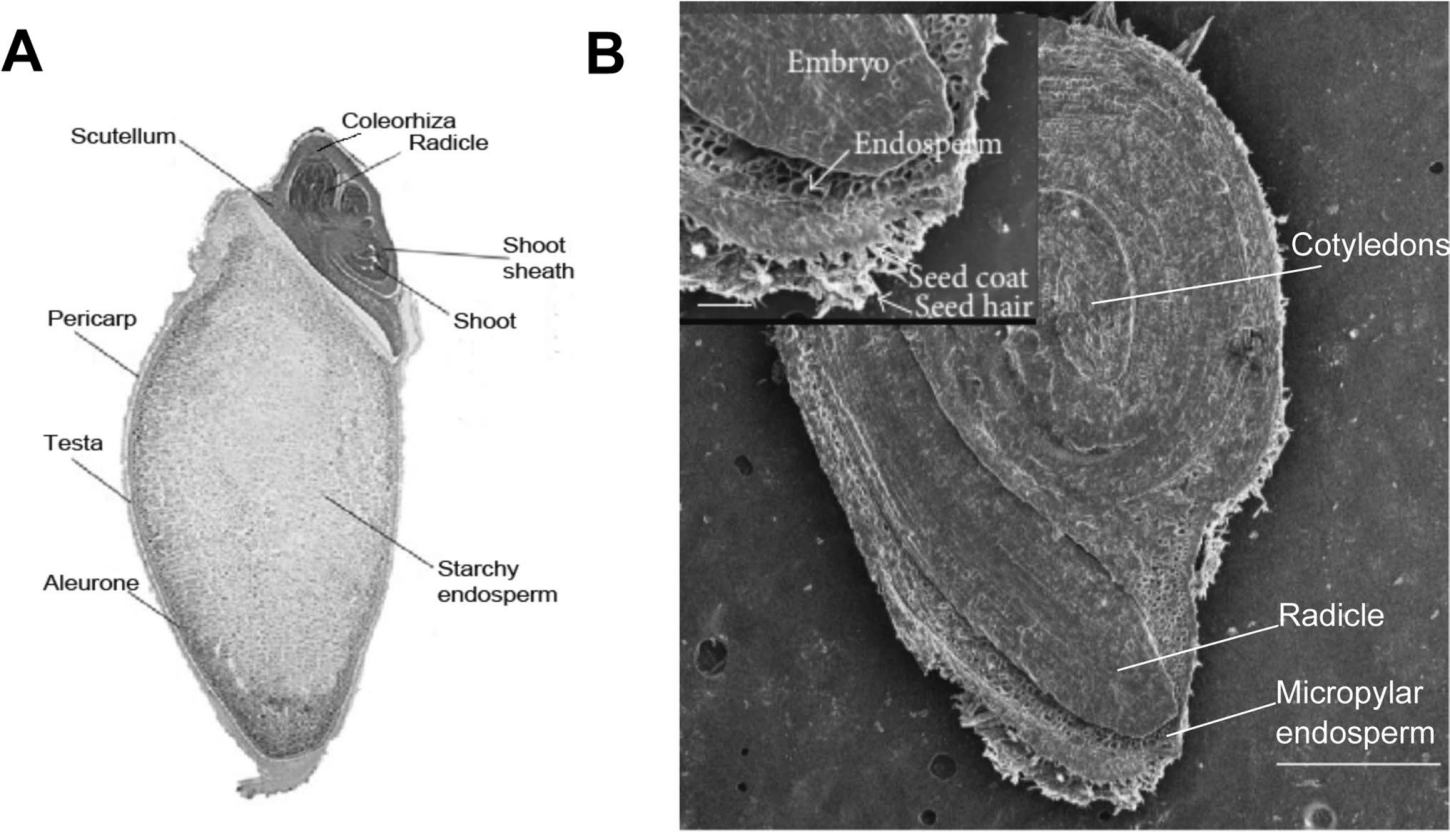
Structures of barley and tomato seeds. **A** Longitudinal section of barley grain (modified from Li et al. 2013). **B** Cross section of a tomato seed as an SEM image. Scale bar in main image is 500 µM and in inset 100 µM (modified from Ratnikova et al. 2015)

The structure of the barley grain is illustrated in Fig. 1A. During germination of cereal grains, GA produced in the epithelium of the embryo scutellum promotes α-amylase expression in the aleurone as well as in the scutellum (Kaneko et al. 2002, 2003; Lenton et al. 1994). α-Amylase and other hydrolytic enzymes expressed in the aleurone are secreted into the starchy endosperm in which they digest starch and other macromolecules to their constituents to be utilised by the growing embryo. α-Amylase genes belong to a large gene family divided into four subgroups, *Amy1-4*, with six *Amy1* genes and three *Amy2 genes* in barley (Zhang and Li 2017). *Amy1* genes encode high pI α-amylases, while *Amy2* encode the low pI isozymes, both subgroups containing GA response elements in their promoters.

As well as inducing expression of hydrolytic enzymes, GA action promotes breakdown of the aleurone cells through programmed cell death (PCD), a process which is prevented by ABA (Bethke et al. 2002). Furthermore, GA and ABA act antagonistically to determine the levels of reactive oxygen species (ROS) in the aleurone, GA causing ROS to accumulate through reducing expression of ROS scavenging enzymes, but the two GA-regulated processes, PCD and ROS accumulation, do not appear to be linked directly (Aoki et al. 2014). The promoters of α-amylase genes contain cis elements consisting of the TAACAAA and TATCCAC pyrimidine boxes forming the GA responsive complex (GARC) that is required for GA-induction of transcription (Gubler et al. 1995). A MYB transcription factor, named GAMYB, was shown by Gubler et al. (1995) to bind to the TAACAAA element in the GARC and promote expression of a high pI α-amylase gene. This group showed subsequently that expression of *GAMYB* is promoted by GA and blocked by ABA (Gubler et al. 2002). In GA-mediated signal transduction, binding to its receptor GID1 enables interaction of the growth-repressing DELLA proteins with an E3 ubiquitin ligase, by which ubiquitination targets them for degradation via the 26S proteasome (Hirano et al. 2008; Itoh et al. 2008). A major function of DELLA proteins is the regulation of transcription, either positively or negatively, by association with transcription factors or transcription inhibitors (Shani et al. 2024). Gubler et al. (2002) showed that GA-induced transcription of *GAMYB* is inhibited by the barley DELLA protein SLN1, but SLN1 did not influence ABA-induced down-regulation of *GAMYB* expression. Expression of *GAMYB* appears to be a primary response to GA action in the aleurone and its transcription precedes that of α-amylase genes, whose expression is a relatively late response (Bethke et al. 1997). Moreover, *GAMYB* expression lags behind that of other GA-induced responses, including changes in intercellular Ca^2+^ concentration, pH and the levels of calmodulin and cGMP (Bethke et al. 1997), but the function of these effects in the response of the aleurone to GA is still unclear.

### Seed germination in tomato is restricted by endosperm integrity

**Fig. 2:**
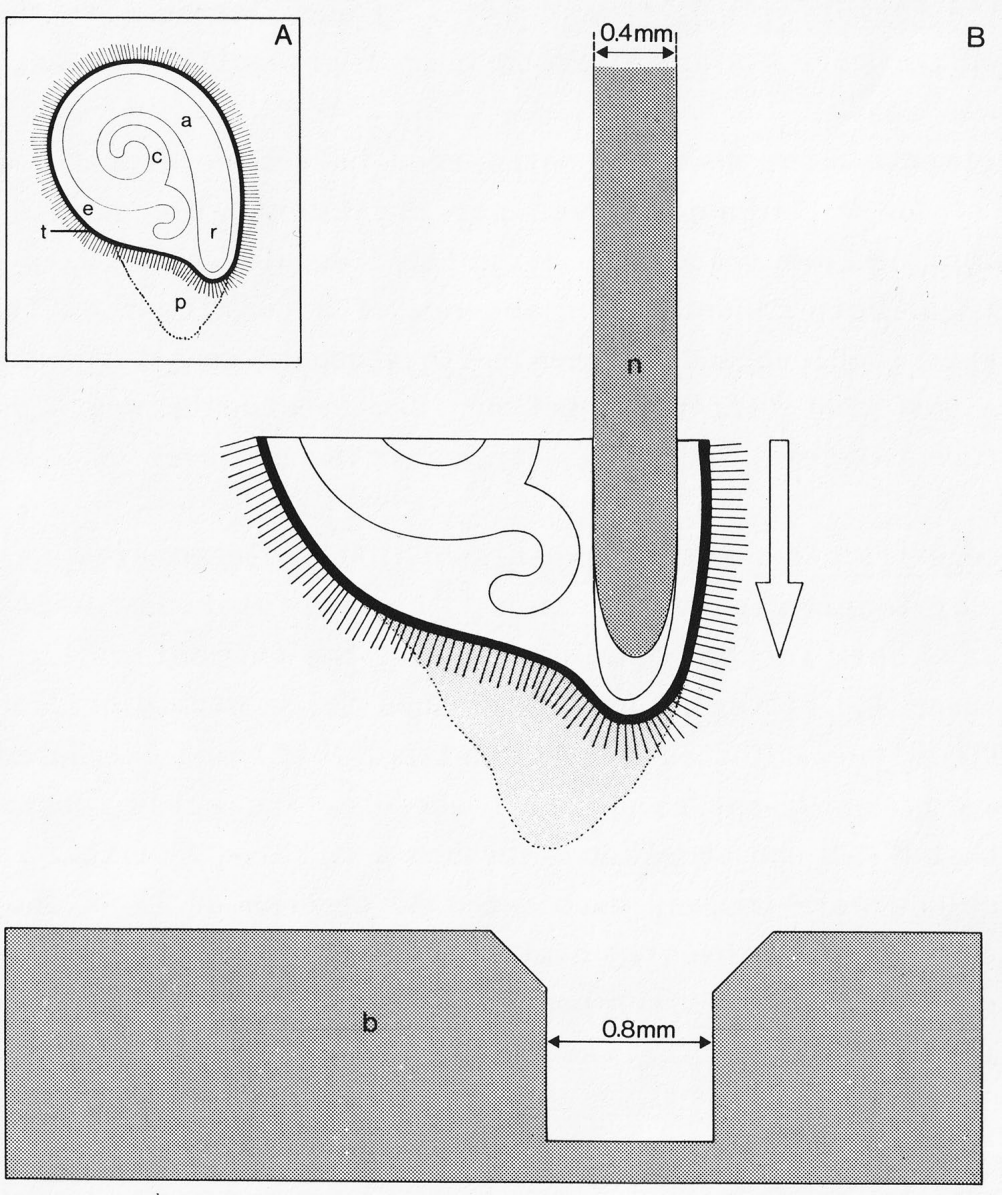
Schematic representation of the method used to measure the puncture force in tomato seeds (original figure taken from Groot and Karssen 1987). **A** Section through a tomato seed showing cotyledons (c), axis (a), radicle (r), endosperm (e), testa (t) and remnants of placental tissue (p). **B** Placental seed-half is shown with the radicle replaced by a needle (n) attached to a tensiometer. The needle plus seed-half move downwards to a block (b) with counter hole

Cereal mutants lacking GAs or GA-responsiveness, while severely dwarfed and infertile, are able to germinate. For example, rice (*Oryza sativa*) mutants lacking GID1 that are completely insensitive to GA are able to form plants with small green leaves (Ueguchi-Tanaka et al. 2005). However, seeds of Arabidopsis (*Arabidopsis thaliana*) and tomato (*Lycopersicon esculentum*) lacking GID1 do not germinate unless the endosperm and testa are removed (Griffiths et al. 2006; Illouz-Eliaz et al. 2019). Plants are then able to form very small green leaves and although they grow very slowly undergo the transition to reproductive development, producing sterile flowers. The promotive effect of GA on germination is well known with GA-deficient eudicots being dependent on an external source of GA (Koornneef et al. 1990; Koornneef and Vanderveen 1980). Groot and Karssen (1987) provided conclusive evidence that in tomato GA promotes germination by weakening the endosperm to enable protrusion of the radicle. Imbibed seeds were cut in half and the embryonic axis was removed, then using a modified needle attached to a precision tensometer they measured the force required to break through the tissues at the micropylar domain opposing the radicle. The method is illustrated in Fig. 2, reproduced from Groot and Karssen. The enclosing tissues consisted of the endosperm, testa and remains of the placenta, but in experiments where just the testa was removed, it was found that the resistance to penetration was due mainly to the endosperm. The tomato seed structure is illustrated in Fig. 1B.

**Fig. 3:**
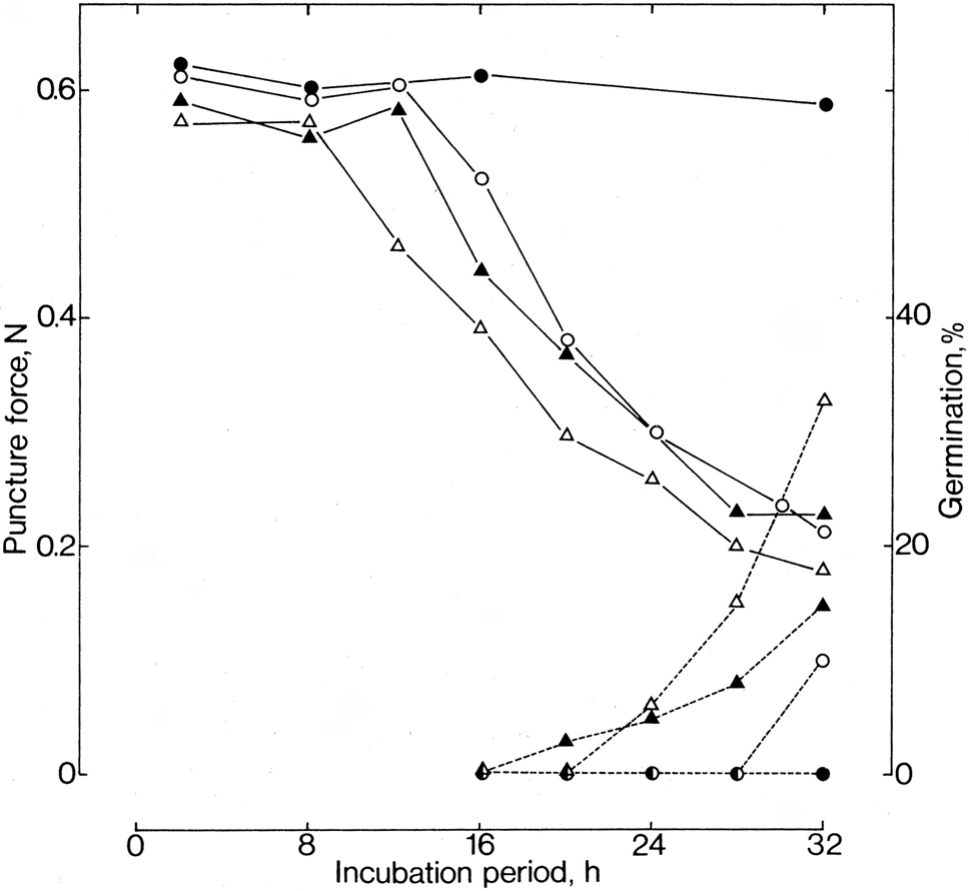
Changes with time of the median force (N) required to puncture the layers opposing the radicle tip (solid lines) and of germination (broken lines) of wild-type (Δ) and *gib-1* (Ο) seeds incubated in water (closed symbols) or 10 µM GA_4/7_ (open symbols). Reproduced from Groot and Karssen 1987

Seeds of the GA-deficient tomato mutants *gib-1* and *gib-2* (referred to by their earlier names of *ga1* and *ga2*, respectively, in the Groot and Karssen paper) failed to germinate unless treated with GA_4_/GA_7_ or GA_3_, with GA_3_ being about 10^3^-fold less effective than GA_4_/GA_7._ Pre-incubation at 2 °C, exposure to ethylene or irradiation with red light failed to promote germination of *gib-1*. However, the fungal metabolite fusicoccin, which promotes cell-wall acidification (Kutschera and Schopfer 1985), stimulated germination of *gib-1* seeds in the absence of GA, although not when the seeds were pre-sterilised with hypochlorite. Germination of *gib-1* occurred in the absence of applied GA if the endosperm and testa adjacent to the radicle were removed, with the plant remaining extremely dwarfed. Groot and Karssen measured the change in force required to disrupt the endosperm/testa layer in the wild type, cv. Moneymaker, and *gib-1* following imbibition. In the first 12 h of incubation in water the force required to break the endosperm/testa layer in the wild type and *gib-1* was about the same at 0.6 N (Newtons), after which it decreased in the wild type to about 0.2 N after 25 h (Fig. 3). The puncture force did not decrease in *gib-1* unless it was incubated with 10 µM GA_4/7_ when it followed a similar decrease profile as the wild type. Treating the wild type with GA_4/7_ reduced the time required to initiate endosperm/testa loosening to 8 h, earlier than for GA-treated *gib-1* seeds. In these experiments the layers were derived from intact seeds, but when experiments were conducted with seeds from which the embryonic axis and radicle were removed after imbibition for two hours, layers from wild type and *gib-1* required the same puncture force which decreased very little during the incubation period, whereas both were reduced with the same kinetics in the presence of GA_4/7_. This suggested that the embryonic axis provided the factor, assumed to be GA, that enabled weakening of the endosperm/testa layer. This was further substantiated by determining the puncture force in embryo-less half seeds incubated for 7 days with isolated embryonic axes. In the presence of wild-type embryos, the required puncture force in both wild-type and *gib-1* half-seeds was 0.2–0.3 N, while with *gib-1* embryos a force of about 0.6 N was required for both genotypes. The authors were not able to confirm that the inductive factor from the embryo was a GA, but in a preliminary experiment an acidic fraction extracted from embryos was found to induce weakening of the endosperm/testa layer.

Since this pioneering paper, the mechanism by which GA action results in weakening of the tissues surrounding the embryo has been extensively investigated, and the findings discussed in numerous reviews, for example (Nonogaki 2014; Steinbrecher and Leubner-Metzger 2017). During germination in tomato and related species, weakening of the endosperm occurs initially in the micropylar region adjacent to the radicle, known as the endosperm cap, while, as reported by Nonogaki et al. (1998) for tomato, post-germination it occurs also in the lateral endosperm. It is associated with the production of cell-wall modifying enzymes, to which endo-β-mannanase is a major contributor in tomato. It occurs in two stages, the first resulting in substantial endosperm weakening which is not inhibited by ABA, while a second ABA-sensitive stage is required to allow germination (Toorop et al. 2000). The mechanism by which GA signalling induces the expression of endo-β-mannanase and other cell-wall modifying enzymes specifically in the endosperm cap is still not completely clear (Nonogaki 2019). Germination depends on the growth of the radicle as well as weakening of the endosperm, both of which are promoted by GA. Martínez-Andújar et al. (2012) reported that many of the transcripts enriched in the endosperm cap during germination were potentially regulated by ethylene, which has been shown to promote germination in some species (Linkies and Leubner-Metzger 2012; Linkies et al. 2009). Furthermore, Martínez-Andújar et al. (2012) found that GA-induction of endo-β-mannanase in isolated endosperm occurred in both the endosperm cap and lateral endosperm with similar kinetics while in intact seeds it occurs initially in the micropylar endosperm. They suggested that in intact seeds, gene expression in the cap could respond to pressure from the radicle through mechanosensing mediated by ethylene. In this case, GA would influence endosperm weakening indirectly through promotion of radicle growth which would explain the spatial specificity of the response.

There has been considerable progress in deciphering the molecular events underlying the regulation of seed dormancy and germination, much of it from work with Arabidopsis, as detailed in a recent review by Carrera-Castaño et al. (2020). While the balance between GA and ABA signalling plays a major role in seed germination, there are contributions from other hormones such as brassinosteroids and ethylene as well as from environmental factors including temperature, light and moisture. Germination in grasses and eudicot species have aspects in common, with GA from the embryo inducing the production of enzymes in the endosperm with eventual endosperm breakdown. While in eudicots the endosperm and in some cases the testa and placenta limit germination by forming a barrier to radicle emergence, in grasses the coleorhiza, an embryonic organ adjacent to the radicle, has a similar function to the micropylar endosperm (Holloway et al. 2021). During germination, the coleorhiza expands and weakens allowing protrusion of the radicle. Germination is associated with induction of cell-wall modifying enzymes in the coleorhiza, a process that is associated with a decrease in ABA, but the involvement of GA is less clear (Banerjee et al. 2014; Barrero et al. 2009; Holloway et al. 2021).

## Conclusion

The work described in the papers highlighted in this article had quite different objectives: in one case the aim was to develop a bioassay for GAs with improved specificity while in the other it was to investigate a mechanism for seed dormancy release in eudicots. Nevertheless, the papers covered related physiological processes, the role of GA in seed germination, and were each groundbreaking, particularly in the development of new experimental protocols that enabled the work that followed. Given the importance of reliable seed germination and seedling establishment to agriculture, the topic is likely to remain at the forefront of plant research. Despite the length of time that has elapsed since these papers were published there is still much to learn on the involvement of hormone signalling in seed dormancy and germination.

## Data Availability

Not applicable.
